# The Temporal Pattern of Arterial Stiffness during Aging: A Large-Scale Cross-Sectional Study

**DOI:** 10.1155/2021/3243135

**Published:** 2021-12-10

**Authors:** Zhengli Tang, Yuanyuan Lu, Yiming Hao, Robert Morris, Di Kang, Fang Wang, Lin Fan, Weijian Wang, Yiqin Wang, Feng Cheng

**Affiliations:** ^1^Shuguang Hospital Health Examination Center Affiliated with Shanghai University of Traditional Chinese Medicine, Shanghai 201203, China; ^2^Department of Biostatistics and Epidemiology, College of Public Health, University of South Florida, Tampa, FL 33612, USA; ^3^Shanghai Key Laboratory of Health Identification and Assessment/Laboratory of Traditional Chinese Medicine Four Diagnostic Information, Shanghai University of Traditional Chinese Medicine, Shanghai 201203, China; ^4^Department of Pharmaceutical Sciences, College of Pharmacy, University of South Florida, Tampa, FL 33612, USA

## Abstract

Brachial-ankle pulse wave velocity (baPWV) is a noninvasive clinical test that provides quantification for the stiffness of both the aorta and peripheral arteries by measuring the brachial and tibial arterial wave velocities. The temporal pattern of baPWV values during aging was investigated in this paper. A gradual increase in baPWV with respect to age was observed, suggesting an increase in the stiffness of arterial vessels as age increases. The *Δ*baPWV value, defined as the absolute value of the difference between bilateral baPWV, also showed a positive correlation with aging. Many underlying physiological conditions such as hyperlipidemia, hypertension, diabetes, and hyperglycemia have previously been shown to elevate baPWV and contribute to the decline of arterial function. The effect of factors including biological sex, blood pressure, and blood glucose levels on the baPWV temporal pattern were also investigated. Between the ages of 18 and 50, men in the study had significantly higher baPWV readings than females of comparable age on average. However, after the age of 50, mean baPWV values increased at a greater rate in females than in males. In addition, blood pressure and blood glucose were shown to be associated with baPWV values. The results will improve existing prediction models for future cardiovascular episodes induced by arterial hardening in different age groups.

## 1. Introduction

The incidence of cardiovascular diseases, and their associated social and economic consequences, is currently on the rise throughout the globe. According to the latest report from the American Heart Association, approximately 3 million deaths were attributed to cardiovascular disease in the United States alone and is the leading cause of death [1]. Globally, approximately 17.8 million deaths were attributed to cardiovascular disease, and as a result of aging populations, it is expected to continue increasing [1]. Previous studies have shown that hardening of arterial vessels is associated with an increased risk of cardiovascular complications and dementia [2–5]. Thus, an early screening of discernable aging of the central or peripheral arteries is an important clinical topic.

Brachial-ankle pulse wave velocity (baPWV) was introduced as an economical measurement for evaluating the mechanical state of the wall of arteries, usually referred as “arterial stiffness,” or rigidity of the arterial wall. It is a noninvasive clinical test that provides quantification for the stiffness of both the aorta and peripheral artery by measuring brachial and tibial arterial waves [6, 7]. Multiple studies have demonstrated the effectiveness of baPWV in measuring the elasticity and hardening of arterial vessels and consequently, characterize the cardiovascular health of cohorts [8, 9]. Large-scale clinical research like the Atherosclerosis Risk in Communities (ARIC) study has used baPWV as an indicator for evaluating arterial stiffness [10]. In addition, pulse wave velocity was found to be associated with other diseases such as frailty. Frailty is a complex physiological state characterized by significant physical and cognitive impairment [11]. A meta-analysis has recently shown a strong correlation between pulse-wave velocity and frailty. Frail individuals exhibit greater PWV readings [12, 13]. Parenthetically, a measure of baPWV has now been incorporated as an option in routine health examinations.

Multiple studies have demonstrated the predictive capabilities of baPWV testing with regard to potential future cardiovascular events. For example, a meta-analysis derived from a Japanese population of *n* = 14,673, of which all participants did not have a history of cardiovascular disease, showed that everyone's standard deviation increase in baPWV (about 385 cm/s) was associated with a 21% increase in cardiovascular disease (CVD) risk [14]. A literature meta-analysis, of which included a large number of individuals (>50%) with medical risk factors associated with CVD risk such as hypertension and late-stage renal disease, showed that every 1 m/s increase in baPWV was associated with a 12% increase in CVD risk [14]. An additional study measured the cumulative incidence of major adverse cardiovascular events (MACE) and showed a strong correlation between elevated baPWV and CVD risk [15]. Thus, many studies have corroborated the effectiveness of using baPWV readings as a predictive indicator of future CVDs.

A healthy baPWV index for individuals of all ages is approximately 1800 cm/s, and an index value greater than this benchmark is associated with an increasingly higher risk of developing cardiovascular disease [16]. Many underlying physiological conditions such as hyperlipidemia, hypertension, diabetes, and hyperglycemia have previously been shown to elevate baPWV and contribute to the decline of arterial function [11–13, 17–20]. Additionally, age and sex have been shown to influence the stiffness of arteries as well as the risk of developing cardiovascular disease [17]. Many factors will contribute to arterial stiffness; however, there is currently no systematic analysis of the association of baPWV with these factors. Previous studies have been limited in their scope and have only analyzed small populations with respect to the association of baPWV with these possible covariates. In addition, those associations at different age groups were not investigated. In this study, we performed a large-scale cross-sectional study composed of more than 6,000 people and analyzed the temporal pattern of baPWV values ranging from young adults to the elderly. In addition, the association between baPWVs and sex, blood pressure, or blood glucose in a multitude of different age groups. Thus, this paper sought to identify the interplay of associations between these possible confounding variables individually with respect to baPWV as a means to fine-tune and improve existing prediction models for future cardiovascular episodes induced by arterial hardening.

## 2. Materials and Methods

### 2.1. Data Source

Our data was collected from participants (between 18 and 87 years old) who went through health examinations in the Shuguang Hospital affiliated with Shanghai University of Traditional Chinese Medicine from March 2018 to February 2019.

### 2.2. Ethics Approval

The study was approved by the Ethics Committee of Shanghai University of Traditional Chinese Medicine and was performed in accordance with the Declaration of Helsinki. All the subjects signed informed consent forms verifying consent and compliance.

### 2.3. Study Design and Data Collection

A retrospective study was performed to investigate the association of physicochemical test parameters and arterial stiffness in adults. All participants underwent anthropometry, health examinations, and blood biochemistry. In addition, the information of each participant's sex, age, and ethnicity were recorded [21].

Anthropometric data were measured using standard methods published by World Health Organization (WHO). The BMI was calculated as body weight divided by height squared (kg/m^2^). The baPWV was measured by an BP-203RPE III instrument (Omron, Co. Ltd.). After the participant rested in the supine position for at least 5 min, a trained investigator wrapped cuffs around both arms and legs and recorded the pulse waveforms from the cuffs simultaneously. The baPWVs were automatically calculated by the instrument. The blood pressure was measured using an HBP-9020 instrument (Omron, Co. Ltd.) in a seated position after a 5-minute rest and was recorded as the mean of two different measurements taken within a 1-minute interval. A fasting blood sample was collected from each participant via the antecubital vein in the morning. Glucose (including fasting plasma glucose (FPG) and hemoglobin A1c (HbA1c)), serum lipids (including total blood cholesterol (TC), triglycerides (TG), low-density lipoprotein cholesterol (LDL-C), and high-density lipoprotein cholesterol (HDL-C)), indicators of liver function (including alanine aminotransferase (ALT), aspartate aminotransferase (AST), and gamma-glutamyl transferase (*γ*-GT)), and indicators of kidney function (including serum uric acid (SUA), serum creatinine (sCr), and estimated glomerular filtration rate (eGFR)) were measured in the hospital laboratory according to routine procedures.

### 2.4. Statistical Analysis

**Figure 1:**
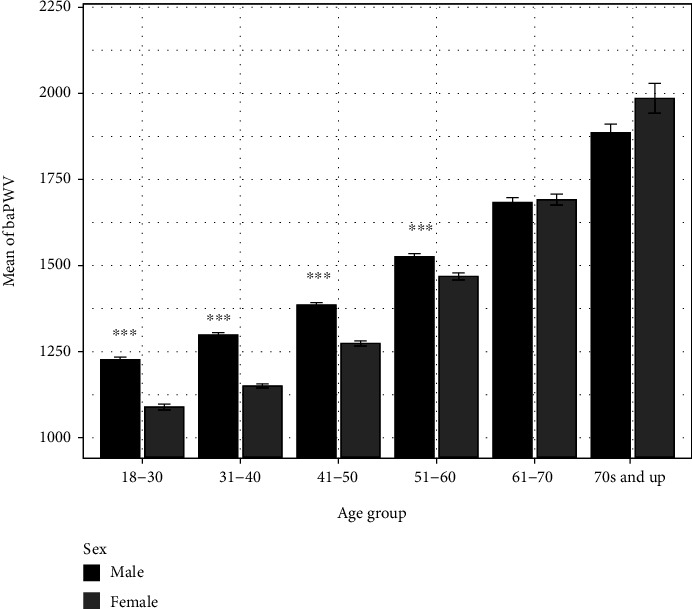
The temporal patterns of baPWV in male and female cohorts. The trend of higher baPWV values in males directly corresponded with age and continued until age 60, of which then the baPWV values of both males and females began to converge. At age 50, female cohorts began to show a faster rate of baPWV elevation. Significance level: *P* < 0.001 (^∗∗∗^), *P* < 0.01 (^∗∗^), and *P* < 0.05 (^∗^).

**Figure 2:**
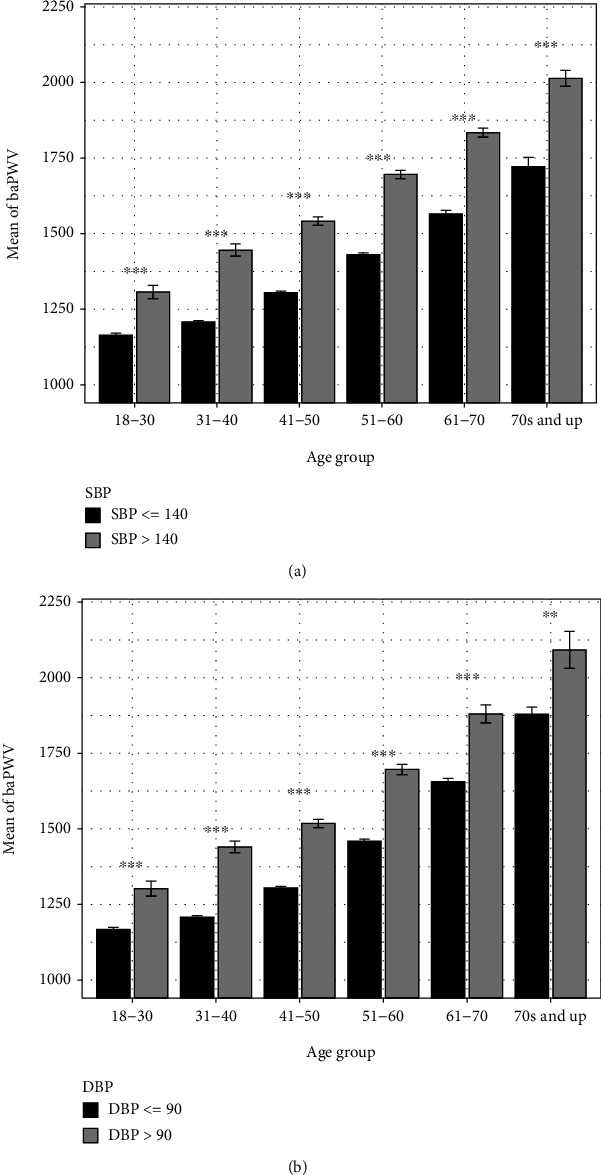
The temporal patterns of baPWV in cohorts with high or low systolic blood pressure (a) and diastolic blood pressure (b). The individuals with raised systolic blood pressure and diastolic blood pressure also exhibited heightened baPWV readings across all age groups. Significance level: *P* < 0.001 (^∗∗∗^), *P* < 0.01 (^∗∗^), and *P* < 0.05 (^∗^).

**Figure 3:**
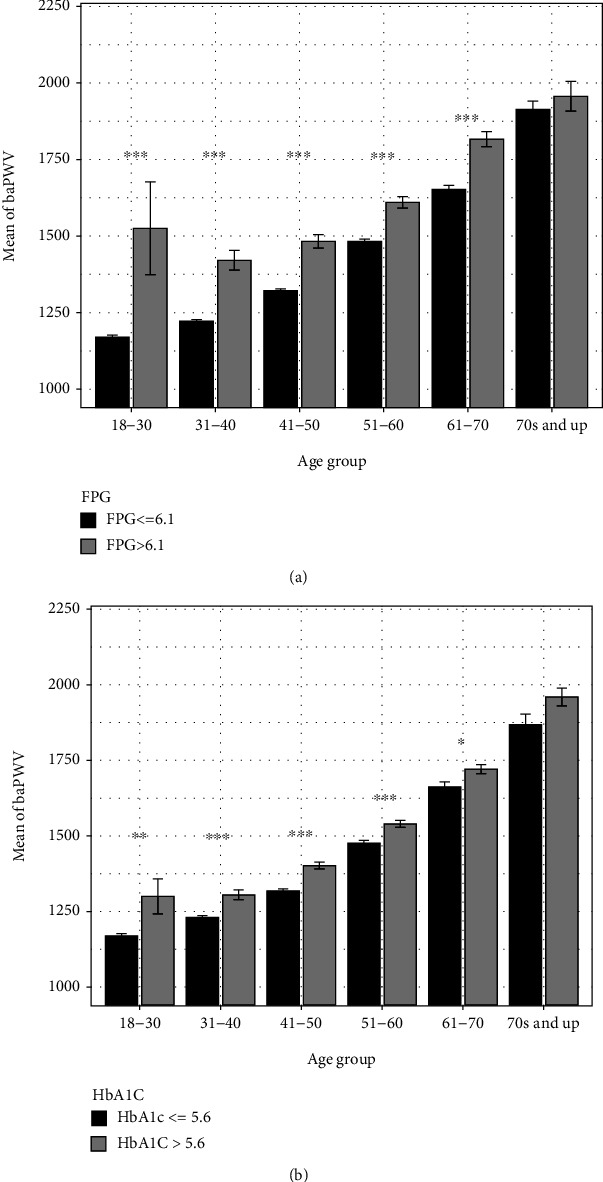
The temporal patterns of baPWV in cohorts with high or low fasting plasma glucose (FPG) (a) and hemoglobin A1C (HbA1c) (b). The mean baPWV for individuals with high blood glucose was significantly greater than the corresponding mean baPWV for individuals with low blood glucose with the exception of those in the “70s and up” age group. Significance level: *P* < 0.001 (^∗∗∗^), *P* < 0.01 (^∗∗^), and *P* < 0.05 (^∗^).

The effect size (Cohen's *d* value) of all continuous variables (shown in Table 1) was calculated using the *effsize* package in R. The group differences (shown in Figures 1–3) were calculated using ANOVA with Bonferroni correction for *post hoc* analysis. All figures were also plotted using the ggplot2 package in R.

## 3. Results

### 3.1. The Demographic Distribution as well as the Results of Health Examinations and Blood Biochemistry Tests

Table 1 summarizes the demographic distribution as well as the results of a series of health examinations and blood biochemistry tests of the sample population. In addition, the mean value of these parameters was compared between individuals with significant arterial hardening (baPWV value ≥ 1800 cm/s) and members of the control group with a control group (baPWV value < 1800 cm/s) by quantifying Cohen's *d* effect size. Based on the magnitude of the resulting Cohen *d* effect size, these parameters can be divided into three groups, large (Cohen's *d* ≥ 0.8), medium (0.8 > *d* ≥ 0.5), and small (0.5 > *d* ≥ 0.2) effect size. Large differences were observed between the control and high baPWV groups with regard to age, SBP, and DBP values. Some factors including FPG and HbA1c showed medium differences in effect size between these two groups.

### 3.2. The Temporal Pattern of baPWV Values

**Figure 4:**
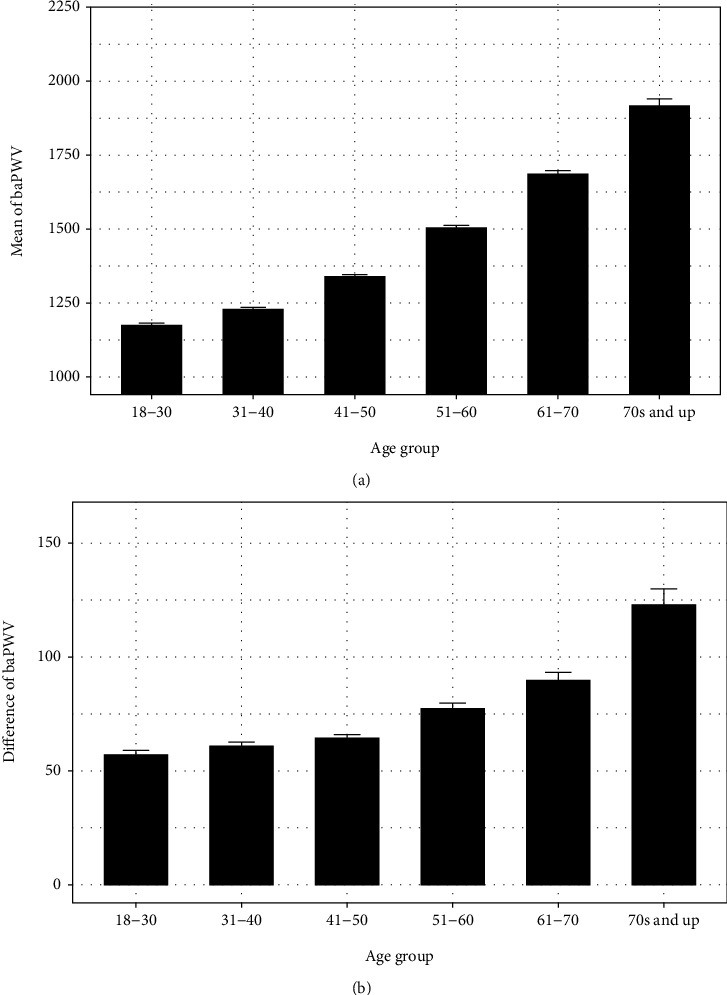
The temporal pattern of baPWV and bilateral baPWV (*Δ*baPWV) values (a) The temporal pattern of baPWV values. A gradual increase in baPWV was observed with respect to age, suggesting an increase in the stiffness of arterial vessels as aging progresses. (b) The temporal pattern of *Δ*baPWV values. The absolute value of differences between left and right baPWV values is referred to as bilateral baPWV (*Δ*baPWV). Similar to baPWV, the values and variances of *Δ*baPWV increase with respect to age.

As Figure 4(a) illustrates, a gradual increase in baPWV was observed with respect to age, suggesting an increase in the stiffness of arterial vessels as aging progresses. Cohorts in the youngest age group, 18 to 30 years of age, exhibited an average baPWV of approximately 1200 cm/s and showed the lowest variance when compared to all other age groups. At age 50, the rate at which baPWV increases steadily rose, and members of these older demographics exhibited greater variance in their baPWV values. In the oldest age group, which included cohort members aged 71 or greater, the average baPWV was approximately 1900 cm/s with variance concurrently being at its peak.

### 3.3. The Temporal Pattern of Bilateral baPWV (*Δ*baPWV) Values

The absolute value of differences between left and right baPWV values is referred to as bilateral baPWV (*Δ*baPWV). The relationship between age and *Δ*baPWV values was shown in Figure 4(b). Similar to baPWV, the values and variances of *Δ*baPWV increase with respect to age. For individuals in the 18-30 age group, the mean *Δ*baPWV was approximately 60 cm/s with a small degree of variance. As age increased, the *Δ*baPWV values and variance nearly doubled in the oldest age group.

### 3.4. The Temporal Patterns of baPWV in Male and Female Cohorts

In the youngest age demographic, males exhibited higher baPWVs than females in the same age group with males having an average baPWV value approximately equal to 1250 cm/s and females having an average of 1100 cm/s. As indicated in Figure 1, this trend of higher baPWV values in males directly corresponded with age and continued until age 60, of which then the baPWV values of both males and females began to converge. After 60, there is no significant difference in baPWV between males and females. At age > 50, female cohorts began to display a more rapid stiffening of arteries than males of the same age group as shown by a faster rate of baPWV elevation. Cohorts of age 71 or higher, for both males and females, had average baPWV values greater than the 1800 cm/s threshold. In addition, males and females exhibited an increasingly wider variance of baPWV values with respect to age with variance reaching its apex in the oldest age group.

### 3.5. The Temporal Patterns of baPWV in Cohorts with High or Low Blood Pressure

Figure 2 show the relationship between baPWV and blood pressure (including the systolic blood pressure (SBP) and diastolic blood pressure (DBP)) in each age group. As shown in Figures 2(a) and 2(b), individuals with high SBP (>140 mmHg) and DBP (>90 mmHg) values also exhibited significantly heightened baPWV readings across all age groups, which indicates a strong correlation between elevated baPWV and heightened blood pressure. In addition, the difference between the mean baPWV of high blood pressure and low blood pressure cohorts increased with respect to age, with the disparity reaching more than 250 cm/s for SBP and 150 cm/s for the DBP in the eldest age group. Despite an increasing trend for blood pressure and baPWV with respect to age, the average baPWV for individuals below the critical threshold for SBP (SBP ≤ 140) did not exceed a baPWV value of 1800 cm/s across all age demographics.

### 3.6. The Temporal Patterns of baPWV in Cohorts with High or Low Blood Glucose

Figure 3 show the relationship between blood glucose level and baPWV values. The fasting plasma glucose (FPG) test is one of the most prevalent diabetes screening methods currently available and measures blood glucose levels after a period of at least 8 hours of no food consumption. Elevated glucose levels during times of fasting are associated with diabetes. Cohorts with an FPG lower than 6.1 mmol/L are considered physiologically healthy while individuals with an FPG higher than 6.1 mmol/L are considered of having some degree of underlying physiological impairment.

As shown in Figure 3(a), in the 18-30 age demographic, the average baPWV value for individuals with an FPG less than 6.1 mmol/L was approximately 1200 cm/s. In contrast, members of the same age cohort with an FPG greater than 6.1 mmol/L had a mean baPWV reading of approximately 1500 cm/s. Thus, there is a strong association between an individual's blood plasma glucose levels and their degree of stiffened blood vessels with respect to baPWV values. However, the difference in baPWV between the high-FPG group and the low-FPG group is highest in the youngest age group and steadily decreases with respect to aging. In the eldest age demographic, there was no significant difference in mean baPWV between individuals with high FPG and individuals with low FPG values. In addition, individuals in the 18-30 age group with elevated FPG levels had the greatest variance in baPWV readings relative to all other age demographics.

As well as the testing for fasting blood glucose levels, measuring levels of HbA1c, defined as the percentage of glucose-bound hemoglobin in the blood, also provides insight into an individual's potential risk for future cardiovascular complications. Healthy individuals are said to have HbA1c levels lower than 5.6%. HbA1c and baPWV were positively correlated with individuals that have elevated HbA1c (>5.6%) concurrently having a greater baPWV reading. In the youngest age (age 18-30) demographic, individuals with physiologically healthy HbA1c levels had a mean baPWV of approximately 1150 cm/s while the mean baPWV for individuals with elevated HbA1c was approximately 1350 cm/s (Figure 3(b)). Like FPG (Figure 3(a)), the mean baPWV for individuals with HbA1c > 5.6% was significantly greater than the corresponding mean baPWV for individuals with HbA1c ≤ 5.6% with the exception of those in the “70s and up” age group. Individuals in the 18-30 age group with elevated HbA1c levels had the greatest variance in baPWV readings relative to all other age demographics.

## 4. Discussion

In this paper, a large-scale cross-section study was performed to identify the temporal pattern of baPWV and bilateral baPWV. The relationship between baPWV and sex, blood pressure, or blood glucose during aging was also investigated.

### 4.1. The Relationship between Age and baPWV Values

**Figure 5:**
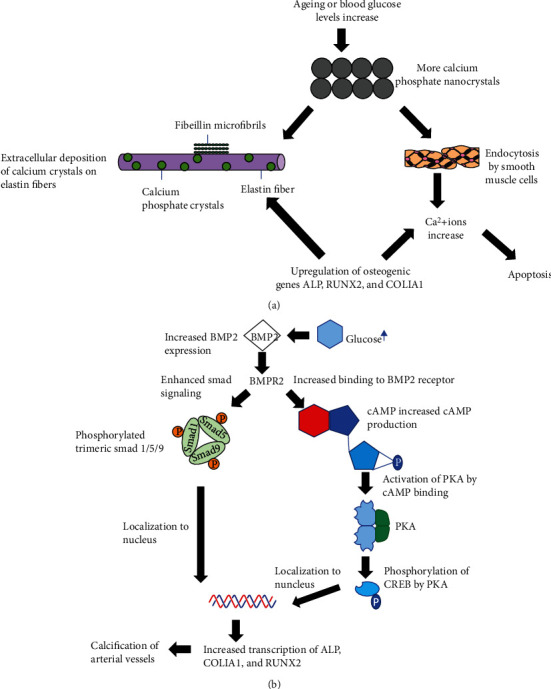
Two calcification mechanisms of action that drive arterial vessel stiffening. (a) Calcification process of arterial vessels induced by ageing or elevated blood glucose levels. (b) Molecular mechanism in which elevated glucose levels promote calcification and higher baPWV readings.

Our results suggested that age is a significant factor associated with artery stiffness with a large effect size (Cohen's *d* = 1.64). Calcification contributes to a more rapid thickening of arterial vessels and subsequently results in the observed elevated baPWV readings during aging [22]. In particular, calcification of coronary arteries (CAC) is influenced by age and sex with approximately 90% of all men and 67% of all women over 70 having some degree of CAC. Two mechanisms of calcification (as shown in Figure 5(a)) may promote this loss of elasticity and drive the pathology of stiffened arteries [23]. The first mechanism involves the extracellular deposition of crystallized calcium and amorphous calcium phosphate on elastin fibers, accelerating arterial stiffness and promoting inelasticity through formation of stress fractures [22]. The second mechanism promotes accumulation of intracellular calcium in smooth muscle cells via upregulated endocytosis of calcium phosphate [22]. Dysregulated intracellular calcium levels facilitate activation of apoptotic pathways and contribute to loss of arterial vessel integrity through the increase of smooth muscle cell death [22, 24]. As a result, disrupted calcium metabolism in old age hastens the stiffening of arterial vessels, leading to higher baPWV readings and greater risk of experiencing an adverse cardiovascular event as age increases [24].

However, some studies such as the IKARIA study, of which assessed aortic stiffness and PWV in a small population known for longevity and great health, showed that the age-related increase in arterial stiffness may be remediated by an increase in physical activity and modifications to lifestyle choices [25, 26]. Other studies have demonstrated an increase in aerobic capacity and a decrease in arterial stiffness in elderly individuals that incorporated moderate physical activity into a previously sedentary lifestyle [27, 28]. Thus, healthy changes in lifestyle choices may protect against the seemingly inevitable age-associated increase in arterial stiffness and dysfunction.

### 4.2. The Sex Difference in baPWV Values

In this study, men were found on average to have considerably higher baPWV values than women of a comparable age up until the age of 50, at which point baPWV began to increase at a faster rate in women relative to men. This may coincide with hormonal fluctuations associated with menopausal and postmenopausal physiology and provide an explanation for the more rapid decline in arterial plasticity found in women in their 60s and 70s. Although the exact mechanism is not clear, estrogen has been shown in some clinical studies to have a cardioprotective role in women and helps to modulate age-dependent stiffening of arterial vessels [29]. In contrast, testosterone has been shown to have a dual effect in which it may promote cardioprotection in some cases while having deleterious effects on cardiovascular health in others [30]. In males, individuals with low testosterone serum levels have exhibited signs of accelerated arterial aging and increased stiffness through higher carotid-femoral pulse wave velocity values (cfPWV) [30, 31]. In populations of men with testosterone deficiency, testosterone supplementation therapy was shown to reverse arterial stiffness to some degree [30]. In females, the significant decline in cardioprotective serum estrogen during and after menopause may, through some yet unelucidated means, promote the anticardioprotective role of testosterone and drive accelerated arterial aging [29, 32]. These hormonal fluctuations may provide insight as to why women experience faster arterial vessel stiffening in old age relative to men of the same age group.

### 4.3. The Relationship between Blood Pressure and baPWV

Based on the effect size, large differences were observed between the control and high baPWV groups with regard to systolic and diastolic blood pressures. Elevated SBP and DBP values, especially for a prolonged period, exacerbate the deterioration of arterial integrity and increases the rate of arterial aging [33, 34]. Healthy blood pressure is maintained by fluctuations in cardiac output and peripheral resistance. However, as one ages, cardiac output declines due to a weakening of healthy cardiomyocyte activity, thus resulting in dysregulation of blood pressure modulation and an increase in mechanical burden on arterial structures [18]. As previously mentioned, prolonged exposure to elevated blood pressure is strongly correlated with an increase in not only cardiovascular disease but also various forms of dementia [35]. One study found a significant increase in A*β* peptide deposition in nondemented patients over time that had higher baPWV and subsequently, severe hypertension [35]. As A*β* peptides are thought to contribute to the development of the cognitive deficits observed in dementia pathology [36], increased arterial dysfunction may be associated with cognitive decline.

### 4.4. The Relationship between Blood Glucose Level and baPWV

In addition to age, sex, and blood pressure, blood glucose levels such as high fasting glucose levels and elevated HbA1c have been shown previously in other studies to be associated with greater baPWV values and higher risk of experiencing an adverse cardiovascular event. In this study, FPG and HbA1c showed medium differences in effect size when assessing their association with baPWV levels.

A recently published observational study (*n* = 2640) following two generations of individuals found a strong correlation between arterial stiffness and markers of hyperglycemia in the parental generation, particularly fasting plasma glucose and HbA1c [37]. Another study suggested that in diabetic patients, high glucose levels were associated with higher baPWV and may promote arterial calcification through an increase in accumulation of intracellular calcium by overstimulation of the BMP-2/RUNX2 signaling pathway [38, 39].  Figure 5(b) summarizes the possible pathway that may explain how elevated glucose levels contribute to greater baPWV values. Hyperglycemic conditions induce bone morphogenetic protein 2- (BMP-2-) mediated signaling by stimulating phosphorylated trimeric SMAD 1/5/9 proteins as well as promoting cAMP production [40]. Upregulation of cAMP signaling leads to an increase in intracellular calcium accumulation while enhanced SMAD activity leads to the upregulation of osteogenic transcription factors such as runt-related transcription factor 2 (RUNX2), alkaline phosphatase (ALP), and collagen type I alpha 1 (COLIA1) [40]. The upregulation of these genes promotes osteoblastic differentiation, of which RUNX2 is an early biomarker of the osteoblast phenotype and propels the calcification of cardiac cells and vascular smooth muscle cells (VSMCs) [38, 41]. It has been shown in a previous study that calcification was inhibited in smooth muscle cells exhibiting RUNX2 deficiency, suggesting a role of RUNX2 in arterial stiffness and promoting elevated baPWV [42]. This phenotypic switch in cardiac cells provides a possible explanation as to why high baPWV values are positively associated with elevated glucose levels and why diabetic individuals are more at risk for experiencing an adverse cardiovascular event.

### 4.5. Limitations

Despite the novelty of the findings, some limitations are present in the study. For example, despite the large sample size, the relative homogeneity of the sample population may limit the applicability of these findings to other populations to some degree. As is the case with cross-sectional studies, the cause and effect between baPWV and some of the analyzed parameters cannot be assessed. This study cannot say with utmost certainty whether elevated baPWV precedes or follows changes in blood pressure, HbA1c, and FPG. However, based on previous studies and review of the literature, it seems more plausible that increases in baPWV are a byproduct of disruptions in these physiological processes and not an initiator of such disruptions. Further studies will seek to better understand the relationship between the confounding factors with one another as well as to dive deeper into the sex-based differences observed in this study. Transcriptomic data will also be analyzed to better understand and confirm the underlying mechanisms of calcification in adults and how various confounding factors influence this process.

## 5. Conclusion

baPWV is a noninvasive and inexpensive means to estimate arterial thickness and approximate future risk of experiencing a major adverse cardiovascular event. Utilizing electronic medical records derived from the Shuguang Hospital in Shanghai, China, between March 2018 and February 2019, a cross-sectional study was performed to assess the age-related temporal pattern of baPWV and bilateral baPWV values ranging from young adults to the elderly. In addition, association between baPWV and additional risk factors previously known to influence the possible risk of adverse cardiovascular events such as sex, blood pressure, and blood glucose in a multitude of different age groups. Age, SBP, DBP, FPG, and HbA1c were found to be significantly associated with baPWV readings. Diabetics or prediabetics may have higher baPWV values due to the induction of BMP-2 by elevated glucose levels. Increased transcription of the osteogenic genes ALP, COLIA1, and RUNX2 may promote calcification of arterial vessels, ultimately leading to stiffened arterial walls and an increase in baPWV. Observed sex differences, particularly with regard to the accelerated stiffening of arterial vessels in women after the age of 50, may be a product of hormonal changes that accompany menopausal and postmenopausal states. The significant reduction in the cardioprotective hormone estrogen may facilitate this heightened decay in arterial integrity and function. Our study provides detailed information for the fine-tuning of existing prediction models for future cardiovascular risk induced by arterial hardening in different age groups.

## Figures and Tables

**Table 1:** The demographic distribution as well as the results of health examinations and blood biochemistry tests.

	Total sample	Low baPWV	High baPWV	Effect size (Cohen's *d*)
Sex, *n* (%)				
Male	3728 (60.1)	3325 (59.5%)	403 (64.8%)	
Female	2482 (39.9)	2263 (40.5%)	219 (35.2%)	
Age (years), mean (SD)	47.9 (12.7), *n* = 6210	46.1 (11.8), *n* = 5588	63.6 (9.4), *n* = 622	1.64
Height (cm), mean (SD)	167.4 (8.4), *n* = 6209	167.8 (8.4), *n* = 5587	164.5 (8.2), *n* = 622	0.39
Weight (kg), mean (SD)	68.5 (12.9), *n* = 6208	68.7 (13.1), *n* = 5587	66.9 (10.9), *n* = 621	0.15
BMI (kg/m^2^), mean (SD)	24.3 (3.4), *n* = 6208	24.3 (3.5), *n* = 5587	24.6 (3.0), *n* = 621	0.10
SBP (mmHg), mean (SD)	128.3 (18.7), *n* = 6182	125.5 (16.4), *n* = 5567	153.9 (18.7), *n* = 615	1.62
DBP (mmHg), mean (SD)	79.3 (11.1), *n* = 6182	78.3 (10.6), *n* = 5567	87.6 (11.7), *n* = 615	0.84
FPG (mmol/L), mean (SD)	5.3 (1.3), *n* = 6180	5.2 (1.1), *n* = 5564	6.0 (1.9), *n* = 616	0.51
HbA1c (%), mean (SD)	5.6 (0.8), *n* = 4889	5.5 (0.7), *n* = 4331	6.1 (1.2), *n* = 558	0.54
TG (mmol/L), mean (SD)	1.7 (1.3), *n* = 6182	1.7 (1.3), *n* = 5566	1.9 (1.4), *n* = 616	0.15
TC (mmol/L), mean (SD)	5.1 (1.0), *n* = 6183	5.1 (1.0), *n* = 5567	5.3 (1.1), *n* = 616	0.14
LDL-C (mmol/L), mean (SD)	3.0 (0.8), *n* = 5566	3.0 (0.8), *n* = 4993	3.0 (0.8), *n* = 573	0.06
HDL-C (mmol/L), mean (SD)	1.3 (0.3), *n* = 5565	1.3 (0.3), *n* = 4992	1.3 (0.3), *n* = 573	0.04
ALT (U/L), mean (SD)	25.0 (19.3), *n* = 6163	25.1 (19.5), *n* = 5554	24.4 (17.6), *n* = 609	0.04
AST (U/L), mean (SD)	23.5 (11.9), *n* = 6163	23.2 (11.7), *n* = 5553	25.6 (13.1), *n* = 610	0.19
*γ*-GT (U/L), mean (SD)	33.8 (32.6), *n* = 6164	33.3 (32.1), *n* = 5554	37.9 (37.1), *n* = 610	0.13
sCr (*μ*mol/L), mean (SD)	73.5 (15.9), *n* = 6189	73.2 (15.5), *n* = 5572	75.8 (19.1), *n* = 617	0.15
sUA (*μ*mol/L), mean (SD)	360.7 (91.9), *n* = 6190	359.8 (91.7), *n* = 5573	368.8 (93.6), *n* = 617	0.10
eGFR (mL/min), mean (SD)	100.0 (17.2), *n* = 5203	100.8 (16.9), *n* = 4702	92.3 (18.3), *n* = 501	0.48

BMI: body mass index; SBP: systolic blood pressure; DBP: diastolic blood pressure; FPG: fasting plasma glucose; HbA1c: hemoglobin A1c; TC: total blood cholesterol; TG: triglycerides; LDL-C: low-density lipoprotein cholesterol; HDL-C: high-density lipoprotein cholesterol; ALT: alanine aminotransferase; AST: aspartate aminotransferase; *γ*-GT: gamma-glutamyl transferase; sUA: serum uric acid; sCr: serum creatinine; eGFR: estimated glomerular filtration rate.

## Data Availability

The Ethics Committee of Shanghai University of Traditional Chinese Medicine limited the measurement data used to support the results of this study in order to protect the privacy of patients. For researchers who meet the criteria for obtaining confidential data, the data of this study can be obtained from Zhengli Tang (e-mail: sgyytzl@163.com).
